# Prolonged, Low-Dose Anti-Thymocyte Globulin, Combined with CTLA4-Ig, Promotes Engraftment in a Stringent Transplant Model

**DOI:** 10.1371/journal.pone.0053797

**Published:** 2013-01-10

**Authors:** Francesca D’Addio, Olaf Boenisch, Ciara N. Magee, Melissa Y. Yeung, Xueli Yuan, Bechara Mfarrej, Andrea Vergani, Mohammed Javeed Ansari, Paolo Fiorina, Nader Najafian

**Affiliations:** 1 Renal Division, Transplantation Research Center, Brigham and Women’s Hospital and Children’s Hospital Boston, Harvard Medical School, Boston, Massachusetts, United States of America; 2 Transplantation Medicine Division, San Raffaele Hospital, Milan, Italy; 3 Divisions of Nephrology and Organ Transplantation, Northwestern University Feinberg School of Medicine, Chicago, Illinois, United States of America; University of Cape Town, South Africa

## Abstract

**Background:**

Despite significant nephrotoxicity, calcineurin inhibitors (CNIs) remain the cornerstone of immunosuppression in solid organ transplantation. We, along with others, have reported tolerogenic properties of anti-thymocyte globulin (ATG, Thymoglobulin®), evinced by its ability both to spare Tregs from depletion *in vivo* and, when administered at low, non-depleting doses, to expand Tregs *ex vivo*. Clinical trials investigating B7/CD28 blockade (LEA29Y, Belatacept) in kidney transplant recipients have proven that the replacement of toxic CNI use is feasible in selected populations.

**Methods:**

Rabbit polyclonal anti-murine thymocyte globulin (mATG) was administered as induction and/or prolonged, low-dose therapy, in combination with CTLA4-Ig, in a stringent, fully MHC-mismatched murine skin transplant model to assess graft survival and mechanisms of action.

**Results:**

Prolonged, low-dose mATG, combined with CTLA4-Ig, effectively promotes engraftment in a stringent transplant model. Our data demonstrate that mATG achieves graft acceptance primarily by promoting Tregs, while CTLA4-Ig enhances mATG function by limiting activation of the effector T cell pool in the early stages of treatment, and by inhibiting production of anti-rabbit antibodies in the maintenance phase, thereby promoting regulation of alloreactivity.

**Conclusion:**

These data provide the rationale for development of novel, CNI-free clinical protocols in human transplant recipients.

## Introduction

The introduction of calcineurin inhibitors (CNIs) into clinical use positively influenced short-term graft survival by reducing acute rejection rates [1]. CNI-containing immunosuppressive regimens are, however, associated with significant morbidity, including infection and neoplasia, whilst CNI-induced nephrotoxicity remains a major barrier to long-term renal allograft survival, and the most common cause of end-stage renal disease in recipients of other solid organ transplants [2], [3]. The achievement of a state of selective hyporesponsiveness towards donor alloantigens whilst maintaining recipient immunocompetence is, therefore, a major goal in clinical transplantation.

The alloimmune response is a complex interplay between pathogenic/inflammatory and regulatory/anti-inflammatory immune mechanisms; the supremacy of either process determines whether the ultimate fate of the allograft is rejection or tolerance, respectively [4]–[6]. Previous studies in renal transplant patients demonstrated that Tregs regulate the alloimmune response and contribute to alloantigen hyporesponsiveness [7], [8]. Various compounds, including Thymoglobulin®, alemtuzumab and sirolimus, are capable of inducing and expanding Tregs *in vitro*, both in animals and humans [9]–[13]. The administration of standard, depleting doses of murine homologue of polyclonal antithymocyte globulin, mATG, has recently been shown to promote the generation of Tregs capable of significant suppression both *in vitro* and in a graft-versus-host disease (GVHD) model [14], and to spare natural Tregs in murine models of skin transplantation and diabetes [15], [16]. Our group first reported that low, non-depleting doses of Thymoglobulin® could expand human Tregs *in vitro* [11], [14], [17], findings confirmed in a kidney transplant study where treatment with low-dose ATG was associated with increased Tregs and donor hyporesponsiveness [18]. We have, furthermore, recently reported that this expansion is dependent on both intact STAT-3 signalling in CD4^+^ T cells and the presence of monocytes [19]; indeed, the addition of ATG to purified CD4^+^ T cells fails to induce any Treg expansion [19], [20].

Preclinical studies demonstrated that B7/CD28 costimulation blockade with CTLA4-Ig effectively inhibits CD4^+^ T cell activation, proliferation and peripheral acquisition of an effector-memory phenotype, thereby reducing graft rejection [21]. Indeed, the recent clinical trials [22]–[25] and FDA approval of a mutant form of CTLA4-Ig (LAE29Y/Belatacept) in human kidney transplantation emphasizes the progression of B7/CD28 costimulation blockade as an immunosuppressive strategy which could avoid CNI toxicity.

We sought to develop a rational preclinical strategy to achieve long-term allograft survival by limiting T cell alloreactivity and expanding Tregs *in vivo*. We utilized a stringent, fully MHC-mismatched murine model of skin transplantation, in which, similar to human transplantation, graft acceptance has been challenging to obtain.

## Results

### A Short Course of CTLA4-Ig with Prolonged, Low-dose and Induction mATG Achieves Long-term Graft Survival

**Figure 1:**
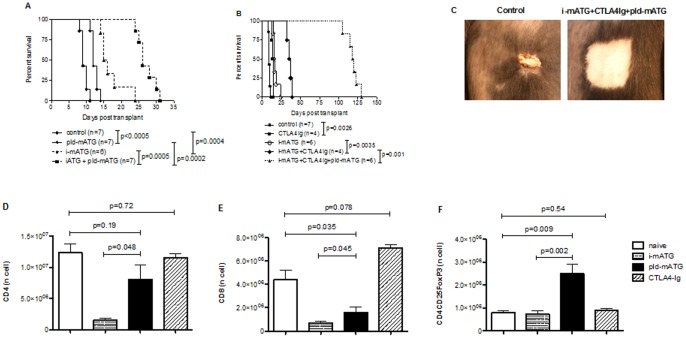
Prolonged, low-dose mATG with a short course of CTLA4-Ig and induction mATG achieves long-term graft survival and favors the emergence of Tregs. **A.** Kaplan-Meier graph depicting fully MHC-mismatched skin allograft survival following the administration of pld-mATG, i-mATG, i-mATG plus pld-mATG, or no treatment. **B.** Kaplan-Meier graphs depicting fully MHC-mismatched skin allograft survival in mice administered i-mATG alone, CTLA4-Ig alone,CTLA4-Ig in combination with i-mATG with/without pld-mATG, or no treatment. **C.** Representative pictures of skin graft from untreated (left panel) and i-mATG plus CTLA4-Ig and pld-mATG-treated mice (right panel). **D/E.** Bar graphs depicting absolute numbers of CD4^+^ and CD8^+^ cells 7–10 days post-transplantation in mice administered either i-mATG, pld-mATG, CTLA4-Ig or no treatment. **F.** Bar graphs depicting absolute numbers of CD4^+^CD25^+^FoxP3^+^ cells (Tregs) 7–10 days post-transplantation in mice administered either i-mATG, pld-mATG, CTLA4-Ig or no treatment. Data are representative of ≥3 independent experiments using ≥3 mice per group per timepoint studied.

In a fully MHC-mismatched skin transplant model, mATG induction (i-mATG; 0.5 mg on days 0 and 4 post-transplantation) alone moderately prolonged allograft survival compared with control Ig, an effect also seen with prolonged, low-dose mATG (pld-mATG; 0.1 mg 2x/week) monotherapy (Figure 1A). The addition of pld-mATG to the i-mATG protocol, however, significantly enhanced graft survival (Figure 1A). While allograft survival was modestly prolonged by a short course of CTLA4-Ig monotherapy (0.5 mg on day 0, 0.25 mg on days 2, 4, 6, 8 and 10), significant enhancement was seen when CTLA4-Ig was administered with i-mATG (MST = 36 *vs* 15.5 days with i-mATG alone, p = 0.0035; Figure 1B). Intriguingly, animals treated with i-mATG plus a short course of CTLA4-Ig with pld-mATG (continued until day 90) demonstrated long-term graft survival, as shown in Figure 1B and C (MST = 119 days). Continued administration of low-dose mATG after day 90 slightly improved allograft survival (MST = 130 days, p = 0.23), but rejection ultimately ensued.

### Prolonged, Low-dose mATG Favors the Emergence of Tregs

**Figure 2:**
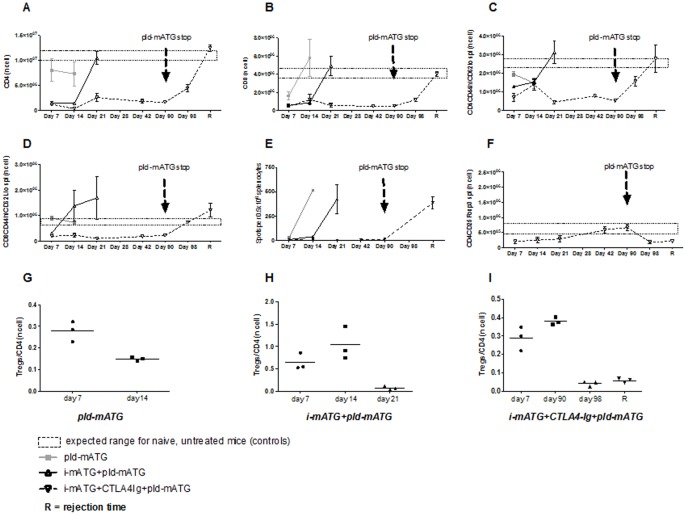
Prolonged graft survival is achieved by inhibiting effector T cells/alloreactive IFNγ secretion and by favoring the emergence of Tregs. A/B. Graphs demonstrating absolute numbers of CD4^+^ and CD8^+^ T cells with various treatment strategies. **C/D.** Frequency of Teff cells (CD4^+^/CD8^+^) using calculated absolute numbers at different timepoints in various treatment groups. **E.** Comparison of donor alloreactive IFN**γ** production at different timepoints after fully MHC-mismatched skin transplantation in animals administered various treatment protocols. **F.** Absolute numbers of Tregs at different time points in mice treated with i-mATG, CTLA4-Ig plus pld-mATG. **G/H/I.** Dot plots representing Tregs/CD4+ ratios in mice treated with pld-mATG alone, i-mATG plus pld-mATGor i-mATG combined with CTLA4-Ig plus pld-mATG during the transplant course. Data are representative of ≥3 independent experiments using ≥3 mice per group per timepoint studied.

We examined the extent of T cell subset (CD4^+^ and CD8^+^) depletion in both spleens and draining lymph nodes (dLN, not shown, similar to spleens) in each of the treatment groups by flow cytometry 7–10 days post-transplantation. As expected, i-mATG resulted in near-complete depletion of CD4^+^ (>85%) and CD8^+^ (>95%) T cells, while pld-mATG caused some depletion of total CD8^+^, but no significant depletion of CD4^+^ T cells compared to naïve controls (Figure 1D–E). Furthermore, Tregs were spared from depletion by i-mATG therapy, while pld-mATG expanded Tregs compared to controls (Figure 1F). CTLA4-Ig did not affect the frequencies of any of the above T cell subsets. Mice treated with i-mATG+CTLA4-Ig+pld-mATG showed complete suppression of both CD4^+^ and CD8^+^ cells compared to controls (Figure 2A–B); repeated enumeration revealed persistent, albeit partial, T cell suppression until the cessation of pld-mATG at day 90, where upon both CD4^+^ and CD8^+^ T cell counts started to recover, precipitating rejection (Figure 1B, 2A–B).

### Prolonged Graft Survival is Achieved by Inhibition of Effector T cells and Alloreactive IFNγ Secretion

We next measured the frequency of overall T effector cells (Teff; defined as CD44^hi^CD62L^low^) by flow cytometry, and of donor-specific alloreactive IFN**γ**-producing splenocytes by Elispot, in the spleens and dLN (not shown, but similar to spleens) of different treatment groups at various timepoints post-transplantation. At 7 days post-transplant, mice treated with i-mATG+CTLA4-Ig+pld-mATG demonstrated a significant decrease in Teff compared to controls (Figure 2C–D), while those treated with pld-mATG maintained a CD8^+^Teff count within the range expected for naïve mice (Figure 2D), indicating that the observed total CD8^+^ T cell depletion (Figure 2B) was mainly due to the elimination of naïve T cells. Although both CD4^+^ and CD8^+^ Teff counts rose from day 14 in the i-mATG+pld-mATG group, eventually contributing to rejection, Teff were suppressed throughout the treatment course in the i-mATG+CTLA4-Ig+pld-mATG-treated group, suggesting a synergistic role of CTLA4-Ig in limiting their expansion over time. The cessation of pld-mATG on day 90 was associated with a progressive rise in both CD4^+^ and CD8^+^ Teff until rejection occurred (Figure 2C–D). Similarly, donor-specific alloreactive IFN**γ** production was completely suppressed in the i-mATG+CTLA4-Ig+pld-mATG-treated group as long as pld-mATG was administered (until day 90), but increased thereafter, accompanied by the recovery of Teff (Figure 2E).

### Treatment with i-mATG+CTLA4-Ig+pld-mATG Favors the Emergence of Tregs

Effector T cell suppression may be achieved by directly affecting Teff, and/or result indirectly from the promotion of Tregs, which inhibit Teff. We therefore next tested the effects of our novel immunomodulatory regimen on Tregs. Samples from dLN (not shown) and spleens from each of the treatment groups were stained for Tregs (CD4^+^CD25^+^FoxP3^+^) 7, 14, 28, 42, 90, and 98 days post-transplantation. In the i-mATG+CTLA4-Ig+pld-mATG group, absolute Treg counts steadily increased until their peak at day 90, whereupon pld-mATG was withdrawn; numbers slowly declined thereafter until rejection ensued (Figure 2F). Using the absolute Treg and CD4^+^ T cell counts at different timepoints, we calculated the Tregs/CD4^+^ ratio (Figure 2G–I), demonstrating a significant increase in the proportion of Tregs over time in the i-mATG+CTLA4-Ig+pld-mATG-treated group until day 90 (Figure 2I; 0.38±0.01), following which it declined, leading to rejection. These data highlight that the achievement of long-term graft survival is associated with tipping the balance of T cell subsets in favor of Tregs.

### Skin Allograft Acceptance Requires the Expansion of Host Natural Tregs (nTregs) by pld-mATG

**Figure 3:**
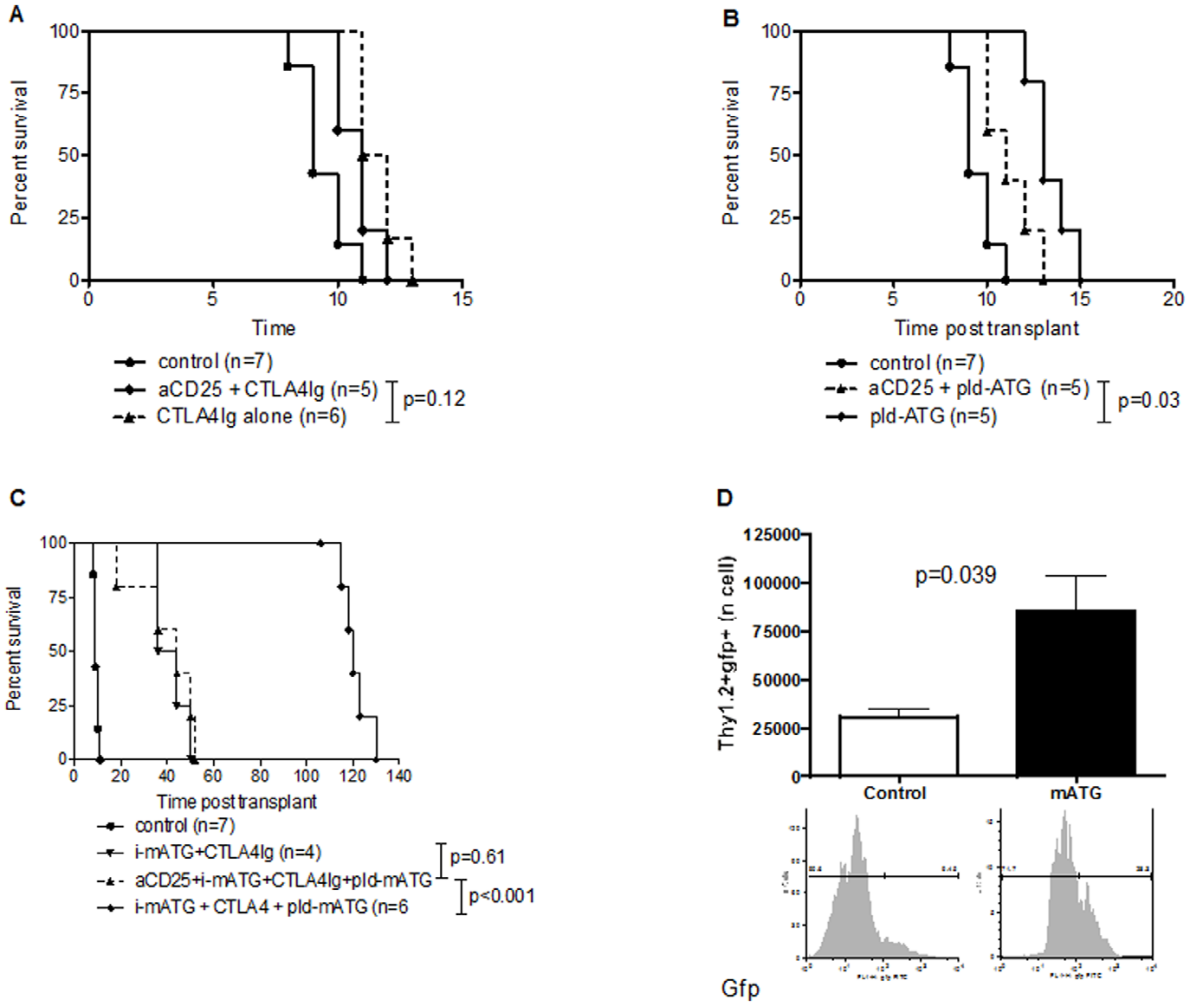
Skin allograft acceptance requires the expansion of host natural Tregs (nTregs) by prolonged, low-dose mATG. A/B/C. Kaplan-Meier graphs depicting fully MHC-mismatched skin allograft survival with or without CD25^+^ cell depletion in mice treated with CTLA4-Ig alone (A), pld-mATG alone (B) or i-mATG, CTLA4-Ig plus pld-mATG (C). **D.** Bar graphs depicting absolute numbers of Thy1.2^+^FoxP3GFP^+^ cells in untreated and mATG-treated groups on day 7. Representative histograms of Thy1.2^+^FoxP3GFP^+^ cells from recipients in each treatment group are shown below the respective bar graphs.

We first evaluated the role of nTregs in long-term allograft survival by investigating the effect of their depletion with anti-CD25 antibody prior to skin transplantation. In control (not shown) and CTLA4-Ig-treated groups, CD25^+^ cell depletion did not affect graft survival (Figure 3A). In contrast, CD25^+^ cell depletion was associated with significant abrogation of graft survival in mice treated with pld-mATG alone, and particularly those treated with i-mATG+CTLA4-Ig+pld-mATG (Figure 3B–C). Intriguingly, the graft survival achieved following CD25^+^ cell depletion in the i-mATG+CTLA4-Ig+pld-mATG group was similar to that achieved by i-mATG+CTLA4-Ig in the presence of CD25^+^ cells but without pld-mATG (MST = 40 *vs* 44 days, p = 0.61), suggesting that the removal of either pld-mATG or nTregs from the protocol has the same effect.

To specifically and directly confirm the role of mATG in nTreg expansion, we studied the effect of mATG therapy or control Ig on allospecific Tregs using an adoptive transfer model. In brief, this is a Thy1.2^+^B6 TCR-tg mouse with CD4^+^ T cells that express a TCR with defined specificity against the bm12 antigen (ABM-tg). GFP-labeled CD4^+^Thy1.2^+^FoxP3^+^ABM-tg T cells are adoptively transferred into congenic WT Thy1.1^+^B6 mice that simultaneously receive a bm12 skin transplant, and are treated with either mATG or control Ig; allospecific cells can then be tracked by the cell surface marker Thy1.2 (CD90.2). The mATG-treated group showed significant expansion of Thy1.2^+^FoxP3GFP^+^ cells compared to controls (p = 0.039, Figure 3D).

### i-mATG+pld-mATG+prolonged CTLA4-Ig Induces Very-long-term Graft Survival, an Effect Primarily Due to pld-mATG

**Figure 4:**
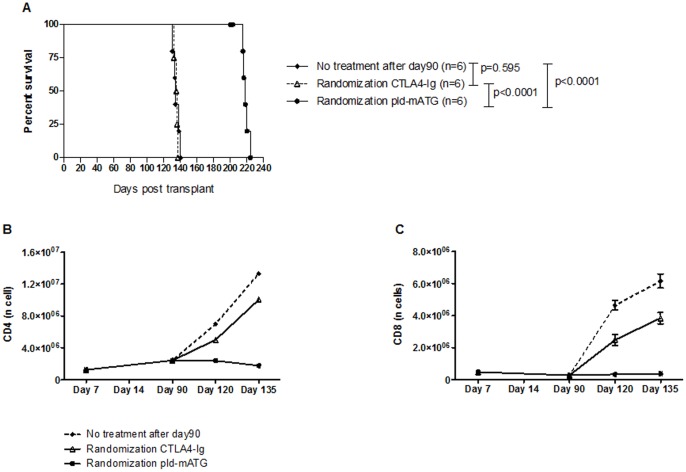
Treatment with i-mATG+pld-mATG+prolonged CTLA4-Ig induces very-long-term graft survival, an effect primarily due to pld-mATG. **A.** Skin allograft survival in mice treated with i-mATG, pCTLA4-Ig plus pld-mATG: on day 90 mice were randomized either to cessation of both treatments or to extended treatment with CTLA4-Ig or pld-mATG alone. **B/C.** Absolute numbers of CD4^+^/CD8^+^ cells at different timepoints post-transplantation in mice treated with i-mATG with pCTLA4-Ig and pld-mATG until day 90 and thereafter randomized to pld-mATG or CTLA4-Ig alone or no further treatment. Data are representative of ≥3 independent experiments using ≥3 mice per group per timepoint studied.

**Figure 5:**
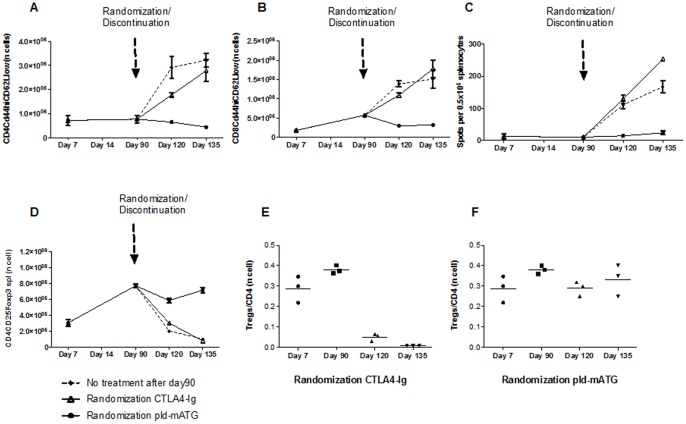
Day 90 randomization to pld-mATG enhances graft survival by favoring persistent Treg emergence.. **A/B.** Frequency of CD4^+^/CD8^+^ effector T cells at different timepoints in mice treated with i-mATG, pCTLA4-Ig plus pld-mATG until day 90 and thereafter randomized to extended pld-mATG or CTLA4-Ig or no further treatment. **C.** Alloreactive IFNγ production at different timepoints in mice treated with i-mATG, pCTLA4-Ig plus pld-mATG until day 90 and thereafter randomized to extended pld-mATG or CTLA4-Ig or no further treatment. **D.** Absolute numbers of Tregs at different timepoints in skin transplant recipients randomized at day 90 to either extended pld-mATG or CTLA4-Ig or no further treatment. **E/F.** Treg/CD4^+^ratios at different timepoints after transplantation in mice treated with i-mATG, pCTLA4-Ig plus pld-mATG until day 90, and thereafter randomized to extended pld-mATG or CTLA4-Ig alone. Data are representative of ≥3 independent experiments using ≥3 mice per group per timepoint studied.

As the combination of i-mATG, pld-mATG and a short course of CTLA4-Ig achieved long-term graft survival, but not tolerance, we next tested the effect of a prolonged course of CTLA4-Ig (pCTLA4-Ig; 0.25 mg 2x/week) in addition to pld-mATG and i-mATG in our stringent skin transplant model. We administered i-mATG and pld-mATG as described, but continued to administer CTLA4-Ig until day 90, whereupon mice were randomized to 3 treatment groups: one group stopped both drugs (no treatment after day 90), another continued only with CTLA4-Ig (randomization CTLA4-Ig), while the last continued with pld-mATG only (randomization pld-mATG). The addition of pCTLA4-Ig to the previous regimen increased graft survival to some degree, although rejection ultimately ensued following the cessation of both drugs on day 90 (MST = 134 vs 119 days, p = 0.003). Intriguingly, the group randomized to continue with CTLA4-Ig alone did not display any further prolongation of graft survival compared to the group in which both treatments were stopped (MST = 135 vs 134 days, p = 0.595). However, the grafts of those animals randomized to continue with pld-mATG survived more than 200 days (p<0.0001; Figure 4A), but ultimately rejected (MST = 218 days). Flow cytometry analysis of dLN (not shown) and spleens on days 90, 120, and 135 demonstrated persistent CD4^+^ and CD8^+^ cell suppression in mice randomized to continue with pld-mATG, while in those randomized to continue with CTLA4-Ig alone, or where both drugs were stopped on day 90, T cell subsets increased until rejection occurred (Figure 4B–C). Similarly, levels of both CD4^+^ and CD8^+^ Teff remained low in the group randomized to continue with pld-mATG, but increased immediately in the other groups following the cessation of mATG on day 90 (Figure 5A–B). Donor-specific alloreactive IFNγ production was completely inhibited by the extended administration of pld-mATG, while the cessation of both drugs on day 90 or randomization to continued CTLA4-Ig alone was associated with a gradual rise in IFNγ production, until rejection ensued (Figure 5C).

### Day 90 Randomization to pld-mATG Enhances Graft Survival by Favoring Persistent Treg Emergence

We demonstrated a persistent increase in Tregs in animals randomized to continue with pld-mATG beyond day 90, while in those randomized to continue with CTLA4-Ig alone, there was a significant decline following the cessation of pld-mATG at day 90 (Figure 5D). Calculation of the Tregs/CD4^+^ ratio demonstrated persistent emergence of the proportion of Tregs when pld-mATG was continued, while in animals randomized to continue with CTLA4-Ig alone, there was a remarkable decline in the proportion of Tregs within the overall CD4^+^ subset (Figure 5E–F). These results confirm that the long-term graft acceptance achieved in the pld-mATG-randomized group is mainly due to the promotion of Tregs within the CD4^+^ population, resulting in an increased Treg/CD4^+^ cell ratio, tipping the balance of the alloimmune response towards regulation.

### CTLA4-Ig Inhibits Production of Anti-rabbit Antibodies, Allowing Unimpeded mATG Function

While randomization to pCTLA4-Ig alone on day 90 did not further enhance allograft survival, continued administration of pld-mATG after day 90 achieved very-long-term graft survival, although grafts were ultimately rejected (MST = 218 days). We then investigated the effect of extended co-administration of pld-mATG and pCTLA4-Ig on allograft survival, and observed indefinite graft acceptance as long as both drugs were administered.

To explore these observations, we investigated if mATG function was eventually affected by the production of anti-rabbit IgG antibodies, considering its prolonged and repetitive administration. We observed high titers of mouse anti-rabbit antibodies in the i-mATG+pld-mATG-treated group (656100 *vs* <100 in naïve serum), confirming that chronic exposure to mATG induces an antibody response. Critically, the addition of either a short or prolonged course of CTLA4-Ig effectively prevented the development of anti-rabbit antibodies (<100 on day 90 and 120). Indeed, cessation of CTLA4-Ig on day 90 and randomization to extended treatment with pld-mATG alone resulted in a high level of antibody production over time (8100 *vs* <100 in naïve serum on day 180) and, ultimately, rejection. The essential role of CTLA4-Ig in preventing antibody production was further evinced by the extended co-administration of pld-mATG and pCTLA4-Ig beyond day 90, which resulted in both graft acceptance and the absence of anti-rabbit antibodies (<100 on day 180).

### Combined Treatment with pld-mATG and CTLA4-Ig does not Inhibit Nominal Antigen Recall Response

Following immunization with ovalbumin (OVA), mice underwent transplantation and either received no treatment or were treated with pld-mATG+CTLA4-Ig for 14 days, whereupon splenocytes were collected and challenged *in vitro* with OVA in an IFNγ Elispot assay. While the number of IFNγ-producing cells increased slightly in the untreated versus treated mice in response to OVA re-stimulation, this did not reach statistical significance (immunized during treatment = 368±49 *vs* immunized untreated = 462±16 spots, n = 3, p = 0.06), indicating that, despite pld-mATG+CTLA4-Ig treatment, the mice remained capable of a nominal antigen recall response.

## Discussion

The tissue toxicity and vascular injury associated with CNI use, in addition to inadequate control of the alloimmune response, are thought to be major barriers to the achievement of long-term graft survival in transplantation. Therefore, the development of strategies to promote allograft survival whilst eliminating the requirement for CNIs, and/or to induce allograft tolerance, have become a major goal in the transplant community [2], [24], [26], [27]. Potential approaches include deletion of peripheral alloreactive effector T cells with induction therapy, inhibition of T cell activation by costimulatory blockade, and promotion of active regulation by harnessing the regulatory potential of Tregs [4], [6], [26]. T cell depleting antibodies successfully prevent acute rejection immediately after transplantation, but recipients ultimately develop subacute or chronic rejection [2], [28]: despite near-complete naïve T cell depletion, a small fraction of T cells are capable of resisting depletion and tend to acquire an effector memory phenotype, undergo homeostatic proliferation and mediate graft rejection [29]. This highlights the necessity of inhibiting homeostatic proliferation of the remaining T cells to establish regulation, wherein two possible strategies have been shown to be effective: costimulation blockade [30]–[32] and Treg exploitation [33]. ATG, long used as a T cell depleting therapy, has more recently been investigated for its potential to expand human Tregs; indeed, our group was the first to report that the addition of low, non-depleting doses of Thymoglobulin^®^ to peripheral blood mononuclear cells (PBMCs) could expand human Tregs *in vitro* [11]. These findings were subsequently challenged by a Canadian study that failed to demonstrate any such expansion using isolated CD4^+^ cells [20]. The use of purified CD4^+^ T cells by the Canadian group is, however, a critical point, as we have recently shown that the *in vitro* expansion of human Tregs by ATG is dependent both on intact STAT-3 signalling in CD4^+^ T cells and the presence of monocytes [19].

Further insights gained from review of the referenced studies formed the rationale to develop a novel therapeutic protocol consisting of T cell-depleting induction, costimulatory blockade with CTLA4-Ig, and prolonged, low-dose ATG. We utilized a stringent, fully MHC-mismatched skin transplant model, where tolerance is difficult to achieve unless released from thymic influence, rendering the continuous restraint of alloreactive effector cells by Tregs essential. In our study, we first utilized mATG as induction therapy (i-mATG), obtaining near-complete T cell depletion whilst sparing Tregs, as previously demonstrated. We then reduced the dose to 20% of that used for induction and administered this for a prolonged period (pld-ATG), demonstrating that, in contrast to naïve mice, this regimen did not suppress the overall CD4^+^ T cell count but did deplete CD8^+^ T cells, although to a significantly lesser extent than the induction dose, and mainly by eliminating the naïve CD8^+^ T cell subset. Crucially, however, pld-mATG significantly expanded Tregs in both the spleen and dLN, and in our adoptive transfer model utilizing FoxP3GFP-ABM mice, expanded alloreactive nTregs. Furthermore, depletion of nTregs abrogated the graft prolongation achieved by the pld-mATG regimen. In aggregate, these data establish the ability of pld-mATG to improve allograft survival by expanding nTregs without significant T cell depletion.

Similarly, the regimen consisting of i-mATG, pld-mATG and a short course of CTLA4-Ig significantly enhanced allograft survival and suppressed effector T cells and the Th1 response over time. Both the absolute number of Tregs and proportion of Tregs within the T cell compartment rose progressively during treatment, thereby tipping the balance in favor of Tregs. Prior Treg depletion and cessation of treatment with pld-mATG, but not CTLA4-Ig, were shown to similarly abrogate the beneficial effect on graft survival in mATG-based treatment groups, suggesting an important role for continuous, low-dose mATG in promoting regulation. These data agree with recent studies by Nador and colleagues, demonstrating the promotion of Tregs by antithymocyte serum in a mixed chimerism tolerance model following combined heart and bone marrow transplantation [34], and by Vergani *et al*, showing improved allograft survival in an islet transplantation model [16]. Our data with OVA immunization emphasize that the combination of pld-mATG and CTLA4-Ig preserves the nominal antigen recall response of the recipient whilst inducing alloantigen-specific hyporesponsiveness.

Finally, the production of mouse anti-rabbit-antibodies was suppressed in combination treatments with CTLA4-Ig, but significantly increased when pld-mATG was administered alone or continued following the cessation of CTLA4-Ig, confirming that CTLA4-Ig inhibits antibody production against rabbit Ig, permitting unimpeded mATG function. This was confirmed by the achievement of very-long-term allograft survival so long as both pCTLA4-Ig and pld-mATG were given concurrently, and by the absence of anti-rabbit antibodies in these mice.

In summary, this study demonstrates that prolonged, low-dose mATG primarily inhibits alloreactive T cells by expanding Tregs, while CTLA4-Ig enhances mATG function by limiting activation of the effector T cell pool in the early stages of treatment, and by inhibiting production of anti-rabbit antibodies in the maintenance phase, thereby promoting regulation of alloreactivity. The combination of prolonged, non-depleting mATG and CTLA4-Ig represents an example of a novel CNI-free regimen so sought in clinical transplantation.

## Materials and Methods

### Mice

C57BL/6 (B6, H-2b), BALB/c (H-2d) and B6.PL-*Thy1^a^*/CyJ (Thy1.1 B6) mice were purchased from The Jackson Laboratory. Thy1.2 FoxP3GFP-ABM TCR-tg mice were generated by breeding FoxP3GFP reporter mice with ABM (anti-bm12) TCR-tg mice which have a TCR that specifically recognizes the MHC class II molecule I-A^bm12^; both parent strains have the C57BL/6 background and bear the Thy 1.2 allele. All mice were used at 6–12 weeks of age.

### Ethic Statement

Mice were housed in accordance with Harvard Medical Area Standing Committee on Animals (HMA IACUC) approval. All the experimental procedures were performed in accordance with Institutional and National Institutes of Health guidelines and Harvard Medical Area Standing Committee on Animals (HMA IACUC) approval. The study was carried out in strict accordance with the recommendations in the Guide for the Care and Use of Laboratory Animals of the National Institutes of Health. All surgery was performed using CO2 overdose euthanasia, consistent with the recommendations of the American Veterinary Medical Association in accordance with Institutional HMA IACUC approval and all efforts were made to minimize suffering.

### Generation and Characterization of mATG

Murine anti-thymocyte globulin (mATG) was generated in a manner analogous to the commercial ATG product (Thymoglobulin®) by immunizing rabbits with a mixture of thymocytes from 8 different strains of mice (C57BL/6, BALB/c, DBA/2, 129, C3H, SJL, Swiss Webster, ICR), as described [9], [35]. Control rabbit IgG was either similarly purified from whole normal rabbit serum or obtained commercially (Sigma, St. Louis, MO).

### Skin Transplantation

Full-thickness trunk skin grafts (1 cm^2^) from BALB/c donors were transplanted onto the flank of B6 recipient mice, sutured with 6.0 silk, and secured with dry gauze and a bandage for 7 days. Skin graft survival was monitored daily thereafter, and rejection defined as complete graft necrosis.

### Treatment Protocols

mATG was administered as induction therapy (i-mATG) at a dose of 0.5 mg on days 0 and 4 post-transplantation; prolonged, low-dose mATG (pld-mATG) treatment consisted of 0.1 mg injected twice a week (2x/week). CTLA4-Ig (Abatacept) was obtained from Bristol-Myers Squibb; the short course consisted of 0.5 mg on day 0 and 0.25 mg on days 2, 4, 6, 8 and 10, while prolonged administration (pCTLA4-Ig) consisted of 0.25 mg 2x/week. CD25^+^ T cell depletion was achieved by treating mice with 0.5mg of anti-CD25 mAb (PC61) on days 6 and 1 pre-transplantation. All treatments were administered by intraperitoneal injection.

### Flow Cytometry

Recipient lymphocytes were isolated from the spleens and draining lymph nodes at various timepoints post-transplantation. Cells were stained with anti-CD3 FITC, anti-CD8 APC, and anti-CD4 PerCP to assess T cell depletion; effector T cells were identified by staining with anti-CD4 PerCP, anti-CD8 FITC, anti-CD62L APC, and anti-CD44 PE (all BD Pharmingen), and analyzed for cells bearing the CD44^hi^CD62L^low^ phenotype. Tregs were identified by staining with anti-CD4 FITC and anti-CD25 PE, followed by intracellular staining with FoxP3 APC (eBioscience), as per the manufacturer’s instructions.

### Transgenic Experiment

Spleens and lymph nodes were harvested from Thy1.2^+^FoxP3GFP-ABM mice; pooled single-cell leukocyte suspensions were prepared. CD4^+^ T cells were purified by negative selection (Miltenyi Biotec, Auburn, CA), achieving>90% purity. An aliquot of cells was stained with anti-CD4, anti-TCR Vα2.1, and anti-TCR Vβ8.1, 8.2, and analyzed by flow cytometry to determine the percentage of ABM TCR-tg CD4^+^ T cells. Typically, >90% of CD4^+^ T cells expressed the tg TCR, and 2% of CD4^+^ T cells were determined to be GFP^+^. CD4^+^ isolated cells were then flow-sorted for GFP^+/−^ cells achieving a purity of 97%. 0.3×10^6^ Thy1.2^+^FoxP3GFP-positive cells were injected intraperitoneally into Thy1.1^+^B6 mice that received a bm12 skin graft and either mATG or control Ig, according to the protocol. On day 7, draining (axillary, lateral axillary) lymph nodes and spleens were collected and Thy1.2^+^FoxP3GFP^+^ TCR-tg T cells enumerated in both groups. To identify the adoptively transferred cells, 1×10^6^ naturally labelled gfp cells were stained with biotinylated Thy1.2 (CD901.2), followed by APC-conjugated Streptavidin. To confirm the cells were indeed all tg, they were also stained with PerCP-conjugated anti-CD4 and both APC-conjugated anti-TCR Va2.1 (B20.1) and PE-conjugated anti-TCR Vb8.1 (MR5-2).

### ELISPOT Assay

Immunospot plates (Cellular Technology, Cleveland) were coated with anti-mouse IFNγ. Recipient splenocytes plus irradiated (30Gy) allogeneic splenocytes (0.5×10^6^ each) were cultured for 24h, incubated with biotinylated anti-mouse IFNγ and streptavidin horseradish peroxidase (DAKO), and developed with 3-amino-9-ethylcarbazole (Sigma-Aldrich). The resulting spots were counted on a computer-assisted immunospot image analyzer (T Spot Image Analyzer, Cellular Technology). All mAbs were from BD Pharmingen.

### Ovalbumin Immunization

Ovalbumin (Sigma Aldrich) emulsified in complete Freund’s adjuvant (Sigma Aldrich) was injected once (100 µg/mouse i.p.) in transplanted mice. Splenocytes were collected and used *in vitro* following rechallenge with 1 µmol/l ovalbumin for 24h.

### Serum Anti-rabbit IgG Titers

Anti-rabbit IgG titers were determined by a standard ELISA procedure. Briefly, microtiter plates were coated with purified rabbit IgG (Bethyl Labs), followed by incubation with serially diluted mouse serum samples. Anti-rabbit antibodies in the serum were detected with an HRP-conjugated goat anti-mouse IgG antibody (Southern Biotech), followed by TMB substrate (BioFX) for 15 minutes; the reaction was stopped with 1N HCL (VWR). Titers were determined as the lowest dilution that averaged above an absorbance of 0.100.

### Statistics

Graft survival was expressed graphically by the Kaplan-Meier method, and statistical differences between groups assessed by the log-rank test. Student *t* test was used for comparison of means. *P*<0.05 was considered statistically significant.
